# 

**Published:** 1894-05-05

**Authors:** 


**Notices of Births, Deaths, and Marriages** are inserted in this column at a charge of 2s. 6d. for an announcement not exceeding four lines.

**Notice to Advertisers and Subscribers.**

All Advertisements, Orders for Copies of The Hospital, and Business Communications should be addressed to **The Publisher, NOT TO THE EDITOR**, The Hospital, 428, Strand, W.C.

The Cost of Subscription, post free, is as follows:—Per week, 2½d.; per quarter, 2s. 8d.; per half-year, 5s. 3d.; per annum, 10s. 6d. Foreign:—Per Annum, 12s. 8d.; payable, in all cases, in advance.

### NOTICE TO CORRESPONDENTS.

All MS., Letters, Books for Review, and other matters intended for the Editor should be addressed The Editor, The Lodge, Porchester Square, London, W.

The Editor cannot undertake to return rejected MS., even when accompanied by stamped directed envelope.

**"Burdett's Hospital Annual," 1894.**

Fifth year of publication. 900 pp. Boards, gilt lettering. A most complete guide to British, Colonial, Indian, and American Hospitals, Dispensaries, Nursing and Convalescent Institutions, and Asylums. Price 5s. Published by The Scientific Press (Limited), 428, Strand, W.C.

**NOTICE.—The Index for Vol. XIV. (ending Sept. 30th, 1893) is on sale at the Office of this Journal, price 2½d., post free.**

### SCRAPS AND GLEANINGS.

The grand concert held in favour of the funds of the Fever Hospital New Ross, proved a brilliant success.

The Christian Endeavour Society continues to hold services in the ophthalmic ward of the Swansea General Hospital.

The Countess Feodore Gleichen has modelled a statue of the Queen, which is to be sculptured in marble, for the Jubilee Hospital at Montreal.

The physicians of the United States now number 118,453. New York leads with 11,171, Pennsylvania has 9,310, and Illinois ranks third with 8,002.

The Central News telegraphs from Rome that Queen Victoria gave a handsome present of money to the Mayor of Florence for the relief of the poor of the city.

It is stated that the late Mr. Matthew Honan, of Cork, has left £40,000 to establish an institute for aged ladies and gentlemen in indigent circumstances.

The Middlesex Hospital has received a donation of 1,000 guineas to endow in perpetuity a bed in the female cancer wards, in memory of Miss Mary Jarret Jacox.

Dr. Bossy, of Havre, whose centenary was celebrated last year, has completed his 101st year. He declares that he is in perfect health and spirits, and mentally and bodily sound.

Mr. Henry Simon, chairman of the Manchester Cremation Society, writes in a contemporary that since the recent starting of the crematorium at Manchester fifty cremations have been carried out there.

A legacy of £50 has been left to the Malvern Rural Hospital by the late Mr. Philip Cofield, and the committee have also had further notice that the late Mr. C. R. Coxwell has bequeathed £100 to the funds.

The total number of new and old cases under treatment at the Birmingham Women's Hospital during the year has been 15,145. Public attention is being drawn to the deficit existing in the finances of the institution.

The total receipts of the bazaar and sale of work held in favour of the funds of the Hull Orthopædic Hospital amounted to £35. The finances of the institution above referred to have been at a somewhat low ebb lately.

The benevolent work of the Home for Protestant Incurables at Cork has been well maintained during the last twelve months. At the close of the year there was left a balance of £166 on the financial working of the institution.

The Pennsylvania Railroad Company has sent out a special train with two physicians, who are to go over the whole line to Chicago and vaccinate at each station all the switchmen, section-men, gatekeepers, and other employés.

The final meeting of those interested in "The People's Mizpah Gift" towards raising £1,000 of the £4,000 required to make the Convalescent Home at Winternsea thoroughly efficient was held recently at the Hull Royal Infirmary. £778 has already been furnished through this means.

During the year 17,586 patients have been treated in the extern department of the Mercy Hospital at Cork. A house adjoining the hospital has been obtained, which will, it is proposed, be converted into a suitable extern department, the upper part supplying additional female wards.

Messrs. Studt and Sons have arranged to hold a grand Venitian fête and gala on June 29th at Swansea, the proceeds of which will be devoted to the funds of the local hospital. It is further rumoured that Madame Patti will arrange definitely to sing for the benefit of the same institution.

A meeting has been held at Stockton, presided over by the Mayor, with the intention of promoting the scheme for the convalescent home for Stockton, Cleveland, and Hartlepool, and the Tee-side district generally. It is proposed to purchase a large mansion and grounds which are suitable for the home.

University College Hospital complains of a falling off of about £100 in subscription, and nearly £700 in donations, coincident with a rising expenditure on general purposes. There is much pressing need for a new hospital building. This, however, cannot be commenced until £30,000 has been collected.

Rhyl Hospital is at length assuming more definite proportions; the Building Committee have been instructed to set to work at once, and the secretary is receiving estimates for the carrying out of the work as soon as possible. The Prince and Princess of Wales have been asked to lay the foundation-stone.

The East London Hospital for Children is earnestly asking for help to meet the heavy increase of the charity's work, 8,500 more patients having been treated last year than in 1892. While the relief thus afforded has been so largely extended, no proportionate addition to the hospital's income has been received.

The last annual report of the North Riding Infirmary stated that the accommodation for patients had been reduced owing to want of money. The increasing pressure of cases for admission have, however, rendered it necessary to withdraw the decision, and the committee are earnestly appealing for further financial support.

By the will of the Right Hon. Hungerford Baron Crewe, the following charitable institutions have benefited by the following bequests: The Chester Infirmary and the Staffordshire Infirmary, £200 each; the North Staffordshire Infirmary, £100; St. George's Hospital, £300; Westminster Hospital, £100; the Seamen's Hospital, Deptford, £100.

The Committee of the Horsham Cottage Hospital report the continued success of the institution, and the generous manner in which its services have been recognised during the past year. The number of patients received in the hospital during which time has been sixty. We congratulate the committee on the excellent manner in which the report has been drawn up.

The Committee of the Meath Hospital and County Dublin Infirmary, in presenting their 142nd annual report, state that the institution in every department continues to be maintained in efficient working order. During the year 172 patients have been admitted into the Bray Convalescent Home in connection with the Dublin Infirmary. The debt balance has increased to £54.

### Medical Vacancies and Appointments.

### APPOINTMENTS.

J. H. Bailey, M.B., Ch.B.Vict., appointed Junior House Surgeon to the Bolton Infirmary. Mr. R. Barnes, appointed Deputy Medical Officer for Barton. Dr. Beamish, appointed Medical Officer to the Newry Fever Hospital. F. M. D. Berry, M.D.Lond., appointed Assistant Anæsthetist to the New Hospital for Women. T. B. Carlyon, M.R.C.S.Eng., appointed Honorary Surgeon to the Tenbury Dispensary. G. A. Cohen, M.B., C.M., appointed Resident Medical Officer to the Hospital for Heart Disease and Paralysis, Soho Square. W. J. Covey, M.R.C.S., L.R.C.P.Lond., appointed Medical Officer to the Infirmary of the Parish of St. Pancras.

### VACANCIES.

The following vacancies are announced this week, the amount of salary in each case being given after the name of the institution:

### Resident Medical Officers.

East London Hospital for Children, Shadwell, E.—House Surgeon. Board and residence provided, no salary. Applications to Thomas Hayes, Secretary, by May 5th.

Flintshire Dispensary.—House Surgeon. Salary £120 per annum, with furnished house, rent and taxes free; also coal, light, water, and cleaning, or, in lieu thereof, £20 per annum. Knowledge of Welsh desirable. Applications to W. T. Cole, Secretary, Board Room, Bagillt Street, Holywell, by May 15th.

Lewisham Union.—Medical Superintendent of the Infirmary; doubly qualified. Salary £275 per annum, with unfurnished house, coals, gas, water, and washing. He will ultimately be appointed Workhouse Medical Officer, at an additional salary of £75 per annum. Applications, on forms to be obtained at the Union Offices, to H. C. Mott, Clerk to the Guardians, Union Offices, 286, High Street, Lewisham, S.E., by May 10th.

Huddersfield Infirmary.—Senior House-Surgeon and Junior House-Surgeon. Salaries £89 and £50 respectively, with board, lodging, and washing. Applications to the Secretary by May 7th.

Parish of St. Leonard, Shoreditch.—Second Assistant Medical Officer for the Infirmary, Hoxton Street; doubly qualified. Salary £40 per annum, with rations, furnished apartments, and washing in the Infirmary. Applications to the Medical Officer, 204, Hoxton Street, N.

### Non-Resident Medical Officers.

Cardiff Union.—Assistant Medical Officer for the Workhouse. Salary £100 per annum, with rations, apartments, attendance, and washing. Applications to Arthur J. Harris, Clerk, Queen's Chambers, Cardiff, by May 10th.

Cardiff Union.—Medical Officer for the Gabalva District. Salary £30 per annum. No extra fees except lunacy. Applications to Arthur J. Harris, Clerk, by May 10th.

Dental Hospital of London and London School of Dental Surgery, Leicester Square.—Demonstrator. Honorarium £50 per annum. Applications to Morton Smale, Dean, by May 14th.

Dental Hospital of London, Leicester Square.—Two Assistant Anæsthetists. Applications to J. Francis Pink, Secretary, by May 14th.

Hampstead Hospital, Parliament Hill Road, N.W.—Dental Surgeon. Applications to the Secretary by May 7th.

London Hospital, Whitechapel, E.—Medical Registrar. Salary £100 per annum. Applications to the House Governor by May 5th.

Vestry of St. Margaret and St. John, Westminster.—Medical Officer; not less than 25, or more than 45, years of age. Salary £250 per annum. Applications, marked on the envelope "Medical Officer," to be delivered at the Town Hall, Westminster, S.W., by May 21st.

Westminster Hospital, Broad Sanctuary, S.W.—Surgical Registrar. Must be F. or M.R.C.S.Eng. Appointment for twelve months. Salary £40 per annum. Applications to Sidney M. Quennell, Secretary, by May 22nd.

